# Vision-Based Steering Control, Speed Assistance and Localization for Inner-City Vehicles

**DOI:** 10.3390/s16030362

**Published:** 2016-03-11

**Authors:** Miguel Angel Olivares-Mendez, Jose Luis Sanchez-Lopez, Felipe Jimenez, Pascual Campoy, Seyed Amin Sajadi-Alamdari, Holger Voos

**Affiliations:** 1 Centre for Automation and Robotics (CAR), Universidad Politécnica de Madrid (UPM-CSIC), Calle de José Gutiérrez Abascal 2, 28006 Madrid, Spain; jl.sanchez@upm.es (J.L.S.-L.); pascual.campoy@upm.es (P.C.); 2 Interdisciplinary Centre for Security, Reliability and Trust, SnT - University of Luxembourg, Weicker 1711, Luxembourg; amin.sajadi@uni.lu (S.A.S.-A.); holger.voos@uni.lu (H.V.); 3 University Institute for Automobile Research (INSIA), Technical University of Madrid, INSIA, Campus Sur UPM, Carretera de Valencia km 7, 28031 Madrid, Spain; felipe.jimenez@upm.es

**Keywords:** road vehicles, vision sensor, automatic steering control, speed assistance, computer vision, vision-based control, positioning, driver assistance vision-based system, partial automation

## Abstract

Autonomous route following with road vehicles has gained popularity in the last few decades. In order to provide highly automated driver assistance systems, different types and combinations of sensors have been presented in the literature. However, most of these approaches apply quite sophisticated and expensive sensors, and hence, the development of a cost-efficient solution still remains a challenging problem. This work proposes the use of a single monocular camera sensor for an automatic steering control, speed assistance for the driver and localization of the vehicle on a road. Herein, we assume that the vehicle is mainly traveling along a predefined path, such as in public transport. A computer vision approach is presented to detect a line painted on the road, which defines the path to follow. Visual markers with a special design painted on the road provide information to localize the vehicle and to assist in its speed control. Furthermore, a vision-based control system, which keeps the vehicle on the predefined path under inner-city speed constraints, is also presented. Real driving tests with a commercial car on a closed circuit finally prove the applicability of the derived approach. In these tests, the car reached a maximum speed of 48 km/h and successfully traveled a distance of 7 km without the intervention of a human driver and any interruption.

## 1. Introduction

Today the concept of autonomous driving is a very active field of research and first solutions of more and more advanced driver assistance systems are already available in recent models of commercial cars. As mentioned in [1], “the majority of the technologies required to create a fully autonomous vehicle already exist. The challenge is to combine existing automated functions with control, sensing and communications systems, to allow the vehicle to operate autonomously and safely”. That report also presents a classification of the level of autonomy based on the capabilities provided by an autonomous system. The simplest system includes the human driver along with an electronic stability and cruise control, which is available in most of the new car models. The next level adds a driver assistance in which steering and/or acceleration is automated in specific situations like parking assistance and adaptive cruise control. The classification then continues with partial autonomy, in which the driver does not control the steering and/or acceleration, but can take over control again if it is required like in lane keeping. After that, there is the level of high autonomy in which the car system is able to operate autonomously in different sections of the journey and only gives the control back to the human driver in some specific dangerous situations. Finally, there is the level of full autonomy in which the vehicle is capable of driving an entire journey without human intervention. Herein, the vehicle must be able to provide all the following specific capabilities:

1. Self-localization in a map or on a predefined path.
2. Sensing the surrounding and identification of potential collisions.
3. Control the basic driving functions, *i.e.*, breaking, accelerating and steering.
4. Decision making, path planning and following while respecting the regulations of traffic.
5. Information collection and exchange, such as maps, traffic status and road incidences.
6. Platooning with other vehicles.

The work presented in this paper is build on the authors previous work [2] which considers a visual line guided system to control the steering of on-board Autonomous Guided Vehicle (AGV) *that pursue a guided path.* This work was initiated with a project done in collaboration with Siemens Spain S.A. focusing on the development of a driver assistance system for buses in the city center using a vision-based line guided system. The main idea was that the driver should still be able to actuate the brake pedal in order to avoid any potential collisions, but has no longer a steering wheel to manually guide the vehicle. Following the previously mentioned level of autonomy, the system presented in this paper could be assigned to a level somewhere between the levels of partial or high autonomy. The presented control system approach takes over the complete control of the steering wheel which corresponds to a high level of autonomy. However, the speed is controlled as an assistance cruise control by keeping the user’s desired speed under the limitation of the maximum speed in each section of the predefined path even if the user pushes the gas pedal to exceed this limit. The speed assistance control also allows to stop the vehicle in case of an emergency, such as the detection of an absence of the line, the push of an emergency button or if a specific localization mark on the road occurs.

The localization of the vehicle on the predefined path was implemented with the help of specific visual localization marks. In cases of a false or missing detection of one or more localization marks, the localization is supported by an odometry approach, i.e. the integration of the speed of the vehicle. These marks were not only used to localize the vehicle, but also to provide additional information to the control system and to the assistance cruise control. This additional information noticeably improves the behavior of the system, such as allowing to reach higher speeds and improving the robustness of the system. Regarding the previous list of the capabilities of a full autonomous system, we are here focusing on Point 1 and partially Point 3. The collision avoidance control is out of scope of this work. Various and potentially adverse conditions of the road such as on rainy days are also not considered in the work at hand. Therefore, the main focus is to find a low cost solution for a vision-based control approach including (I) the steering of an autonomous vehicle using a line guide, (II) a speed control assistance and (III) the localization of the vehicle, while preserving robustness against brightness variations in inner-city environments.

The layout of the paper is as follows. Section 2 discusses related works on autonomous cars. Section 3 describes the full system architecture as well as the low-level car controller (Section 3.2), and the human-machine interface developed to remotely supervise the system, to command the desired speed and to realize an emergency stop if needed (Section 3.3). Section 4 presents the derived computer vision algorithms. Section 5 presents the general system architecture, the car automation and the control approach of the steering wheel. The results of experiments that were carried out in a closed test road are presented in Section 6. Finally, Section 7 presents the conclusion and the future work of this paper.

## 2. Related Works

Autonomous guided vehicles (AGVs) are generally used in manufacturing and logistic systems inside warehouses, but their acceptance inspired many other applications such as guided buses in city transportation. They were introduced during 1950s and, by 1960s the Personal Rapid Transit (PRT) began to advent. Different guidance systems were introduced for AGVs such as systems based on optical distance measurements, wires, magnetic tapes or computer vision. Each type is based on own design requirements and comes with own related advantages and disadvantages. For instance, in wire guidance systems, a wire is installed below the floor on which the AGV is moving. The wire emits a radio signal which can be detected by a sensor on the bottom of AGV close to the ground. The relative position to the radio signal is applied by the AGV to follow the path. In magnetic tape guidance system, a flexible tape of magnetic material is buried in the floor or road like in the case of the wire guidance system. The advantage of this method with respect to the wire guidance system is the fact that it remains unpowered or passive. In laser navigation systems, the AGV is equipped with a laser transmitter and receives the reflection of the laser from a reflective tape installed on the surrounding walls. The localization and navigation is done using the measurements of the angles and distances to the reflectors. However, this method is generally only used in indoor environments [3,4]. A vision based navigation system uses a vision sensor to track landmarks in the environment which means that no magnets, no induction wires and also no laser technique is required to let the AGV follow a specified path [5,6].

On the other hand, the motivation to reduce traffic jams, to improve the fuel economy and to reduce the number of vehicle accidents in transportation leads to the introduction of different levels of automated driving. Many research institutes and automotive manufacturers worldwide are introducing their automated driving solutions, based on proprioceptive sensors such as the Anti-lock Brake System or the Electric Stability Program, or based on exteroceptive sensors such as radar, video, or LiDAR [7]. The very first experiments on autonomous vehicles have been started in 1920s and promising steps have been conducted in the 1950s. The research in autonomous driving in Europe started within the PROMETHEUS project (program for a European Traffic with Highest Efficiency and Unprecedented Safety) which was one of the largest research projects in fully automated driving in 1986. The obtained results of this project are regarded as milestones in the history of vehicular and robotic systems. Two of the vehicles were ARGO by VisLab [8,9] and VaMoRs [10] tested in 1998. Both of them used two cameras to detect road lanes and to avoid obstacles, but the implemented algorithms and strategies were different. In 1995 the NAHSC project (National Automated Highway System Consortium) started in the United States within the California PATH (Partners for Advanced Transit and Highways) program [11]. In 1997, the important Demo’97 was developed in San Diego in which some cars were guided by a magnetic guided line inside the asphalt. An array of different sensors had been installed in those cars to execute self-driving tests and to form automated platoons of 8 cars.

In the last decade many authorities around the world introduced plans to promote the development and establishment of automated vehicles [12]. Numerous commercial vehicles offer some levels of automation, such as adaptive cruise control, collision avoidance, parallel parking system, lane keeping assistance, *etc*. Research on this topic got a strong impulse by the challenging test-bed of DARPA in the grand and the urban challenge in 2005 and 2007 [13] with impressive results obtained by Sebastian Thrun and his team from the Stanford University in 2005 [14] and 2008 [15], or by the Braunschweig University in 2009 [16]. All of these works tried to cover all the capabilities listed for a fully autonomous system, which is also the case for the recent Google Car [17]. In this specific case, the obtained results of this approach should enforce legal changes to achieve the first license for a self-driving car. The European Union also has a long history of contributing to automated driving such as the Vehicle and Road Automation (VRA) program, the GCDC (Grand Cooperative Driving Challenge), and others. Many countries plan to develop sensors, control systems and services in order to have competitive autonomous driving systems and infrastructures [18]. A considerable number of studies and projects have been funded or are still continuing within the new HORIZON2020 research framework in the European Union.

For instance, a Mercedes-Benz S-Class vehicle equipped with six radar sensors covering the full 360° angular range of the environment around the vehicle in the near and far range has been introduced in 2013. The vehicle drove completely autonomous for about 100 km from Mannheim to Pforzheim, Germany, in normal traffic [19].

Moreover, there are also some works focusing on the sixth point of the list of autonomous capabilities (*i.e.*, the platoon formation). In 2010, the multidisciplinary European project SARTRE used new approaches in platoon formations and leader systems to successfully present an autonomous platooning demo traveling 120 miles [20]. The platoon comprised one human-driven truck followed by four cars equipped with cameras, laser sensors, radar and GPS technology. A complete test of different systems of leader following, lane and obstacle detection and terrain mapping has been done by the VisLab. In 2010, the laboratory directed by Alberto Broggi covered the distance of 15.926 km from Parma to Shanghai with a convoy of four motor homes [21,22]. All of them were equipped with five cameras and four laser scanners, no road maps were used. The first vehicle drove autonomously in selected sections of the trip while the other vehicles were 100% autonomous, using the sensors and the GPS way-points sent by the leader vehicle. The control of speed and/or steering of autonomous vehicles with a localization system based on GPS information is also presented in the literature, see, e.g., [23]. Herein, a cruise control approach for an urban environment comprising the control of the longitudinal speed based on the speed limits, the curvature of the lane and the state of the next traffic light is proposed. In [24], control tests of a high-speed car running the Pikes Peaks rally drive are presented. The work in [25] shows a localization system without GPS, based on the detection of intersections and the use of a virtual cylindrical scanner (VCS) to adapt the vehicle speed.

Highly automated levels of driving require a very wide range of capabilities like sensing the environment, figuring out the situation and taking proper action for the driver. The design of a cost-effective solution for such highly automated driving systems is challenging and most of the time leads to an expensive multi-sensor configuration like the way introduced in [26]. Vision-based systems are considered to be a cost-effective approach for automated driving systems [27]. Vision-based systems can be categorized in different research areas and applications in the field of automated driving such as distance estimation using stereo vision [28,29] or monocular vision data [30]. A review of the literature in on-road vision-based vehicle detection, tracking, and behavior understanding is provided in [31]. From an algorithmic point of view, computationally more complex algorithms require an understanding of the trade-off between computational performance (speed and power consumption) and accuracy [6,32]. For instance, an offline-online strategy has been introduced in [33] to overcome this trade-off. Furthermore, vision-based systems have many applications in automated driving, such as road detection which is one of the key issues of scene understanding for Advanced Driving Assistance Systems (ADAS). In [34] road geometries for road detection are classified, and [35] introduces an improved road detection algorithm that provides a pixel-level confidence map. The paper [36] describes a neural network road and intersection detection. Another vision-based application for ADAS is lane keeping assistance, where a technique for the identification of the unwanted lane departure of a traveling vehicle on a road is presented in [37].

Despite some new and improved computer vision algorithms which have been introduced in recent years such as [38], it has to be noted that the variation in the lighting conditions, occlusions of the lane marking or road shoulders, and effects of shadows make the current vision-based solutions not reliable for the steering control of an autonomous car. Furthermore, these algorithms are still not completely real-time capable to be used in the closed control loop. Based on that and the specific constraints of our project mentioned in the previous section, this work focuses on a vision-based line guided system approach. To the author’s best knowledge, a vision-based line-guided system has not been presented to control the steering of an autonomous car under the maximum speed constraints of urban environments.

## 3. System Description

In this section the system description of the automated car is presented, which can be divided in the general system architecture, the vehicle automation and the human-machine interface.

### 3.1. General System Architecture

**Figure 1:**
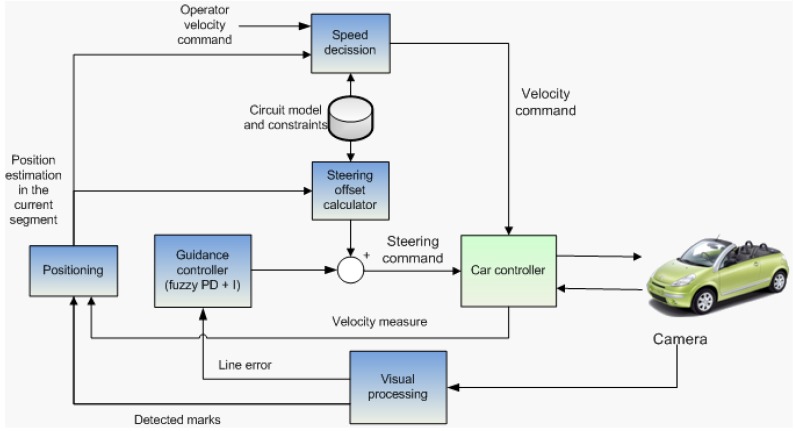
Full system architecture.

The system architecture comprises several components as depicted in Figure 1. The first component is the computer vision system (described in Section 4), also called “Visual processing”, which generates the information of the local position of the vehicle with respect to the path to follow (painted as a line). In addition it is able to detect binary visual marks painted on the road, which have the encoded information of its global positioning on the track as well as the maximum speed inside this section and the curvature radius. This information is stored in a data base.

The lateral control system described in Section 5 includes the “guidance controller”. These are two feedback controllers which have to keep the vehicle on the path by minimizing the deviation from the path. This component also includes the “Steering Offset”.

A “positioning” system allows to calculate the position of the vehicle on the track. The odometry information is generated by integrating the vehicle’s speed and updated with the absolute information obtained by reading the visual marks. In case a mark is not detected at the begining of one section, the odometry position estimation is used to change from one section of the track to another.

The “speed decision” system limits the speed of the vehicle by taking its position on the circuit into account. Herein the human driver is not permitted to overwrite this information either manually or using the Human-Machine Interface (HMI, see Section 3.3).

Both velocity and turning commands are sent to the “car controller” system (see Section 3.2), which actuates directly with the motors installed in the vehicle and reads the measured speed of the vehicle.

### 3.2. Vehicle Automation: Car Controller System

**Figure 2:**
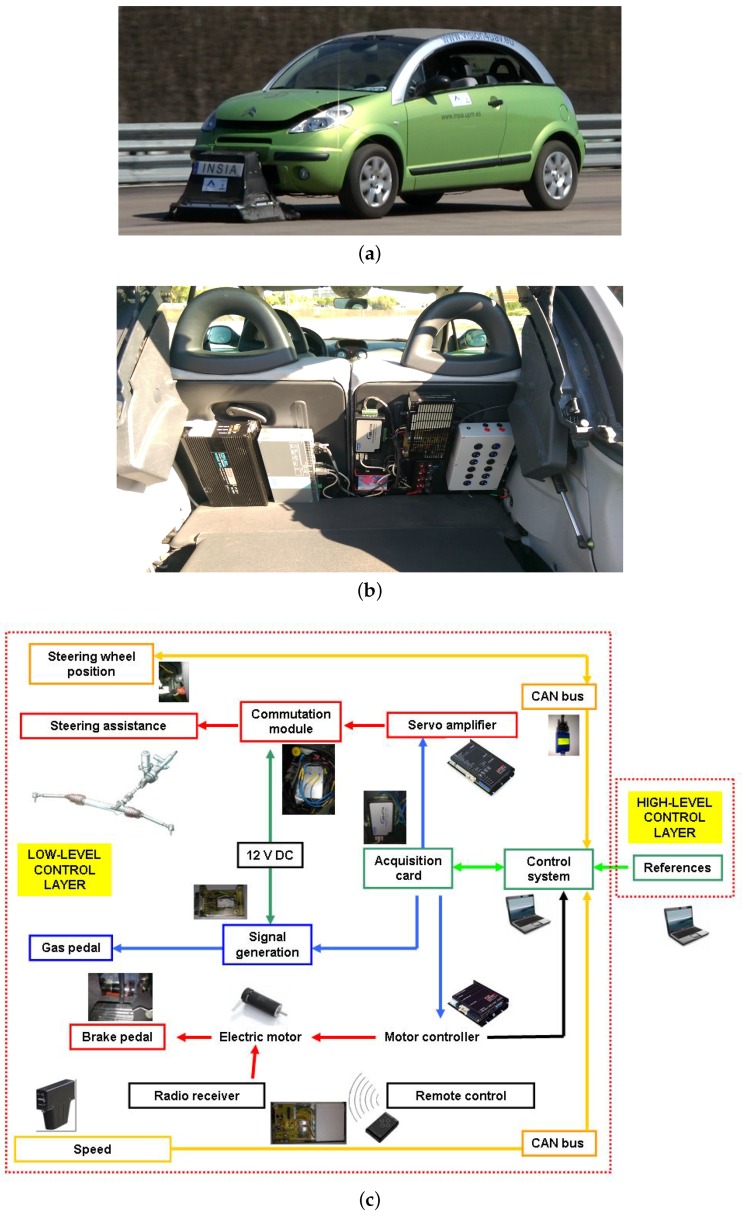
Passenger vehicle automation. (**a**) Autonomous vehicle; (**b**) electronic deployment of the control system; (**c**) internal architecture for vehicle control.

Within the project considered in this paper, a passenger car (shown in Figure 2a) has been fully automated. A detailed description of the vehicle automation is included in [39] and the steering and speed fuzzy controllers were described and tested in [40]. The automation includes a control unit that communicates with a data acquisition card that provides signals to the electronic accelerator pedal and to a servo amplifier operating on the vehicle steering assistance motor. This solution is applicable in case of vehicles with an electric steering assistance system. In the case of hydraulic assistance, solutions as shown in [41] could be used. Moreover, the control unit sends commands to the electric motor controller which operates the brake pedal. As a safety measure, a remote control could operate on this motor so that any control signal is blocked and an emergency braking signal is provided from the power source. The fuzzy controllers allow, firstly, a vehicle behavior similar to that of a human driver. In addition, functions such as emergency manoeuvres (emergency brake and steering avoidance manoeuvres respecting conditions of stability) which are generated if an obstacle is detected on the route have been implemented in the decision module [39]. Figure 2b shows the assembly of the electronic control units of the automated vehicle. Moreover, this low-level control layer receives the desired signals of steering wheel angle and vehicle speed from a high-level control layer and Figure 2c shows an overview of the architecture of the internal automatic control of the vehicle.

This architecture allows the implementation of different types of applications such as a GPS-trajectory guided vehicle, line tracking, collision avoidance applications, remote control from a mobile device like a smartphone, *etc*.

### 3.3. Human-Machine Interface for the Driver Supervision

**Figure 3:**
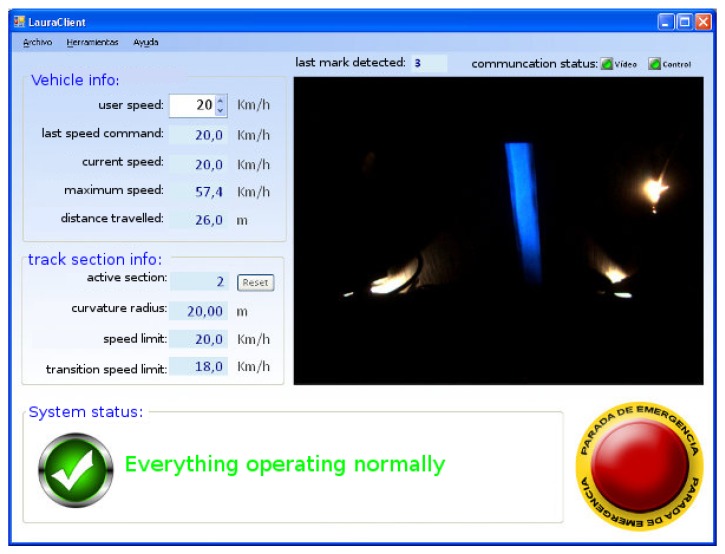
Developed human-machine interface.

In this work, an autonomous driving system is presented which is always under the supervision of a driver, either a remote external operator or a driver inside the vehicle. For that reason the presented HMI for the supervision task was designed, see Figure 3. It shows the current information of the vehicle (“*vehicle info*”), comprising the desired user speed (“*user speed*”) that can be changed in real time, the current desired speed (“*last speed command*”), the current speed (“*current speed*”), the maximum speed reached during all the experiment (“*maximum speed*”), and the distance traveled in the current section of the track (“*distance traveled*”). Below this information, the information related to the current section of the track in which the vehicle is traveling (“*track section info*”) is shown. The first line shows the id of the current section (“*current section*”), followed below by the curvature radius of the current section of the track (“*curvature radius*”), the speed limit inside the section (“*speed limit*”), and the limit for the transition between the previous section and this section (“*transition speed limit*”). Any system messages appear at the bottom of the HMI as a state of the system (“*System status*”), e.g., a message indicating a breakdown of the communication or any camera error is shown in this field. On the right side of this bottom part of the HMI, an emergency stop button (yellow and red button) was also installed. Once this button is pushed, the vehicle will stop whatever its state is. A real-time image feedback of the camera is also shown in the HMI. Over the image feedback, the id of the last mark detected (“*last code detected*”), the state of the camera, and control communications (“*communication status: video, control*”) is shown, respectively. Both communications are managed with a specific software daemon to reconnect autonomously after the detection of a communication breakdown.

## 4. Computer Vision System

A computer vision algorithm processes in real time the images captured by a monocular camera under illumination with ultraviolet (UV) light. This camera is placed in the front part of the vehicle, looking downwards and isolated from the sunlight by a black box. A similar approach with a looking downward camera at the bottom of the car is presented in [42]. In this work the car structure was used to avoid that the illumination changes affect the image acquisition. The camera was used to get information from the road to do a localization matching between vision based ground features and the global localization done with a RKT-GPS system. No control of the steering wheel was presented in this work. We, in the presented work, have to set the camera on the front of the vehicle since we are controlling the steering wheel in an Ackermann model vehicle. The mentioned approach could be used in our case to get the information from the visual marks painted on the road but not to guide the vehicle.

The presented algorithm detects both the line to be followed by the vehicle, as well as visual marks painted on the road. The visual marks provide a coded information associated to forward path properties like curvature, maximum allowed speed, *etc.*, which is used by the controller to anticipate changes and react faster.

**Figure 4:**
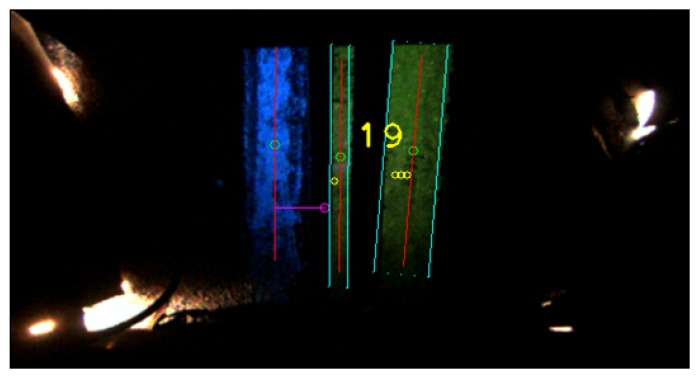
Line and mark detection by the computer vision algorithm.

Two kinds of paint were used for the line and the visual marks. Due to their different pigments, the line is seen as blue while the marks seen as yellow in the images captured by the camera under illumination with UV light. The rest of the image remains black, as depicted in Figure 4.

The visual algorithm has been designed with a special focus on robustness and thus being able to detect fragmented lines due to occlusions or small irregularities on the road. The full system has been tested under different weather conditions including sunny and cloudy days as well as sparkling days. To evaluate the robustness of the system it is important to know the exactly speed at which the system is able to see every single cm of the track by the camera installed. To know this value it has to be taken into account that the frame rate of the camera is equal to 29 fps and the size of the camera system (the metallic box) is 30×50 cm. The distance covered by the system is equal to 29 fps×30 cm=870 cm/s, and 870 cm/s are equal to 31.32 km/h. That means that at this speed the captured frames cover all the track without losing any single cm of the road. When the speed of the vehicle is higher than 31.32 km/h the system will lose some distance covered by the vehicle in between each frame captured. In the case of 40 km/h, which is equal to 1131 cm/s, the system cover a distance of 39 cm per frame, and for 50 km/h (1388.8 cm/s) the system cover a distance of 47 cm per frame. That means that the system can not see 9 cm and 17 cm in between each frame for the speed of 40 and 50 km/h respectively. Despite this limitation of the vision system the vehicle was able to detect the line and visual marks covering successfully long distance at different speeds. The computer vision algorithm has two different parts which will be described in the following sections.

### 4.1. Line Detection

This first part of the visual algorithm processes the current acquired image to obtain information about the line to be followed by the vehicle. If there is a line in the analyzed image, the line angle and distance are determined with respect to the image center.

The first step for the line detection is color segmentation on YUV space that exploits the blue appearance of the line in the image. Some other color spaces were tested, but YUV provided better results under different light conditions. A rectangular prism inside the YUV space is defined so that only the pixel values inside this volume are considered part of the line. The output of this first step is a binary image in which only the line pixels are set. This method proved to be robust in detecting lines of different blue tones and brightnesses.

To reduced the noise, a second step is performed. In the binary image, every 8-connected pixel group is marked as a blob. Blobs having an area outside a defined range are discarded. Then, for every survivor the centroid, the dominant direction and the maximal length are computed. Those blobs with a too short maximal length are ignored. The remaining blobs are clustered according to proximity and parallelism, so each cluster becomes a candidate line. The centroid and dominant direction of each candidate line are calculated from the weighted sum of the features of its component blobs, where the weight of each blob is proportional to its relative area. In this way the algorithm can accurately detect lines that are fragmented because of the aging of the paint.

The last step consists of the choice of the winning detected line from the whole set of candidate lines. The decision is achieved by using temporal information between the current and the previous frame, *i.e.*, the candidate closer to the last frame winner in terms of centroid distances will be selected as the current frame winner. This rejects false positives because of old line traces along the circuit. In the case that all candidates are far enough from the last frame winner, a bifurcation is assumed and the winner will be the leftmost or rightmost candidate, depending on the information associated to the last detected visual mark.

### 4.2. Mark Detection and Decoding

The second part of the computer vision algorithm includes the detection and decoding of visual marks painted on the road next to the line to follow. The visual marks are detected and decoded even when they appear rotated in the image as a result of vehicle turns.

Each visual mark is labeled through a unique identifier that represents a binary-encoded number, where mark bits are drawn as bars parallel to the line. Because of the reduced visual field of the camera, instead of painting a bar for each bit like in common barcodes where the width of the bar depends on the bit’s value, a more compact encoding scheme was chosen. All bits are bars with the same width with no spacing between them. When a bit is one, the bar is painted; when it is zero, the bar space is left unpainted. In the image a mark appears as a set of yellow rectangles. Every painted rectangle is a bit with value one.

A start bar is added at the furthest bit slot from the line to designate the beginning of the mark. The mark specification also defines the number of bits per mark, a fixed bit width, a minimum bit length, and valid ranges for line-mark angle and line-to-start-bit distance. According to the specification, the algorithm will only detect marks that are placed on the right of the line in the direction of motion of the vehicle.

Similarly to the line detection phase, the mark detection algorithm follows a set of steps. First of all, the acquired image is segmented by color in YUV space, extracting the potential mark pixels. The color space boundaries of this segmentation are set so that it admits several yellow tones, ranging from tangerine yellow to bright green, as seen in tests with multiple paints. This makes the color-based filter less restrictive and avoids false negatives. As the probability that any yellow noise present in the image has a valid mark structure is low, the following steps of the algorithm seek for this valid structure in the color-segmented pixels to reduce the false positives.

After the color segmentation, the resulting eight-connected pixels are grouped in blobs. The blobs that do not meet the following criteria are considered as noise and are discarded: the blobs must appear at the right of the line, the blob area must be in a valid range (computed from the visual mark specification), the angular distance between the dominant blob direction and the line must be in the specified range, and the blob length in the dominant direction must be larger than the minimum bit length.

The blobs that pass the filters correspond to a set of bits with value 1. A pattern matching determines the specific ordinal number of bits for each blob. Assuming each mark has a total of *N* bits (including the start bit), *N* pattern tests are carried out for each blob, one test for each bit in the range [1,N]. For every bit *i*, the pattern $P_{i,B}$ is a rectangle with the direction and length of the blob *B* and a width *a* equal to *i* times the bit width in the specification (in pixels). The ordinal number of bits for the blob *B* will be the value *i* that minimizes the cost function present in the Equation (1).

(1) $$c\left(i,B\right)=f\left(i,B\right)+\hat{g}\left(i,B\right)$$

where

$$\begin{array}{c} f\left(i,B\right)=1-\frac{a(B\cap P_{i,B})}{a(B)}\\ \hat{g}\left(i,B\right)=\frac{g(i,B)}{g_{max}}\\ g\left(i,B\right)=|a\left(B\right)-a\left(P_{i,B}\right)| \end{array}$$

and a(S) is the area in pixels inside the shape *S*.

Function *f* indicates how much the pattern covers the blob. Function *g* evaluates the similarity between the blob and pattern areas. Patterns whose *f* or *g* are above a threshold are discarded. This forces the best solution to have a minimum quality in both indicators. Then, the minimization process favors patterns that cover the blob while having a similar size. The threshold for *g* is $g_{max}$ and $\hat{g}$ is a normalized version that stays in [0,1], like *f* does.

After assigning a number of ones to all processed blobs, the rightmost one is interpreted as the start bit. If its distance to the line is in the range allowed by the specification, the mark remains in the detection process, otherwise it is ignored. The mark’s dominant direction is computed as the average of all its blobs. The orthogonal vector to this direction defines a baseline that is divided into *N* consecutive fixed-width bit slots, starting from the start bit. All bit slots are set to an initial value of zero. Then, blob centroids are projected on the baseline and each projection falls into one of the slots, an then filled with a one. Adjacent slots are also filled with ones according to the blob’s number of bits. Finally, the slot values define the binary-encoded mark identifier whose least significant bit is the closest to the start bit.

**Figure 5:**
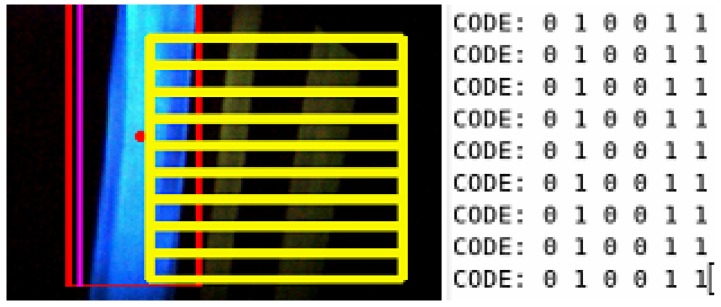
Single frame voting process for the detection of visual marks.

The final detection step of the visual mark identifier that is going to be passed to the control system is elected with a two different voting process. The first voting process evaluates the detected visual mark id in each frame. Working under the assumption that the code is always located on the right of the line, this part of the image is divided in nine horizontal sections. In each of these section the system tries to identify a visual mark. The final result comes from the most detected code. An example of this voting process is shown in the Figure 5.

The second voting process evaluates the code detection among the last *M* frames. Large values of *M* produce higher detection delays but increase detection robustness, as more image samples are taken into account. In our experiments, M=3 gave good results. Besides the detected mark identifier, the algorithm provides an estimation of the number of frames and time lapse since the mark was last seen. This information is especially useful at high speeds and high values of *M*, when the decision is delayed until *M* frames have been captured, but the mark was only seen on the first few frames. In addition, an estimation of the mark quality is given based on its average luminance. Figure 4 shows the detection of the line and the mark which represent the number 19.

Once the mark detection stage provides a visual mark identifier, the decoding stage starts. The information encoded in these marks is the current location of the marks on the track, the size and the curvature of the following section of the track, and the maximum permitted speed on it.

The detection of a mark is always checked with a database of available marks, avoiding false detections that could localize the vehicle in another section of the track.

## 5. Lateral Control System: A Vision-Based Fuzzy-Logic Controller

**Figure 6:**
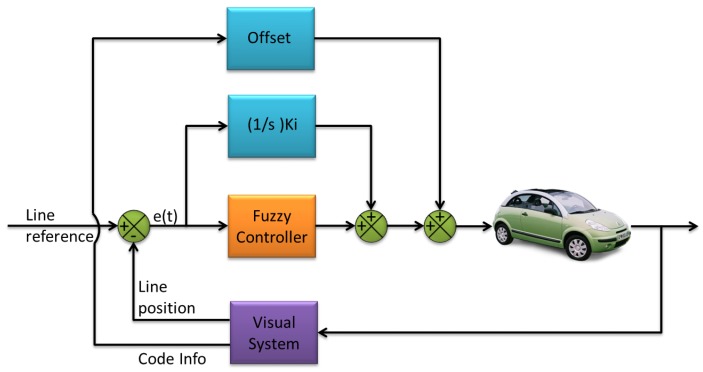
Simplified control loop of the visual control system.

The steering control system of the vehicle includes three additive components: The first one is a fuzzy-logic feedback controller that acts with a behavior equivalent to a PD controller. The second one is the weighted integral of the error (distance between the line reference and the measured line position). The third component is the steering offset that acts like a feedforward controller, by changing the operating point of the fuzzy-logic controller to improve its performance, based on the track information given by the detected mark. All the three components are added at the end of the control loop to generate the output of the control system, making a structure of Fuzzy+I+Offset, as shown in Figure 6.

The objective of this work is to develop a controller for a track with small radius curves. In such conditions the speed of the car is not allowed to be very high (less than 50 km/h).

From several real experiments with the vehicle, the authors can confirm that it is practically impossible for a human pilot using just the information received from the down-looking camera to drive faster than 10 km/h while keeping the line-following error low enough to meet the requirements of the application. This is because the pilot only sees 39 cm ahead, and, at that speed, the contents of this area change completely every 0.108 s.

The first and main component, the fuzzy-logic feedback controller was implemented using a software called MOFS (*Miguel Olivares’ Fuzzy Software*). This C++ library has been previously successfully used to implement fuzzy-logic control systems with other kind of robotic platforms such as an unmanned helicopter for autonomous landing [43] or quadrotors for avoiding collisions [44]. Thanks to this software, a fuzzy controller can be defined by specifying the desired number of inputs, the type of membership functions, the defuzzification model and the inference operator. In [43], a more detailed explanation of this software is provided.

**Figure 7:**
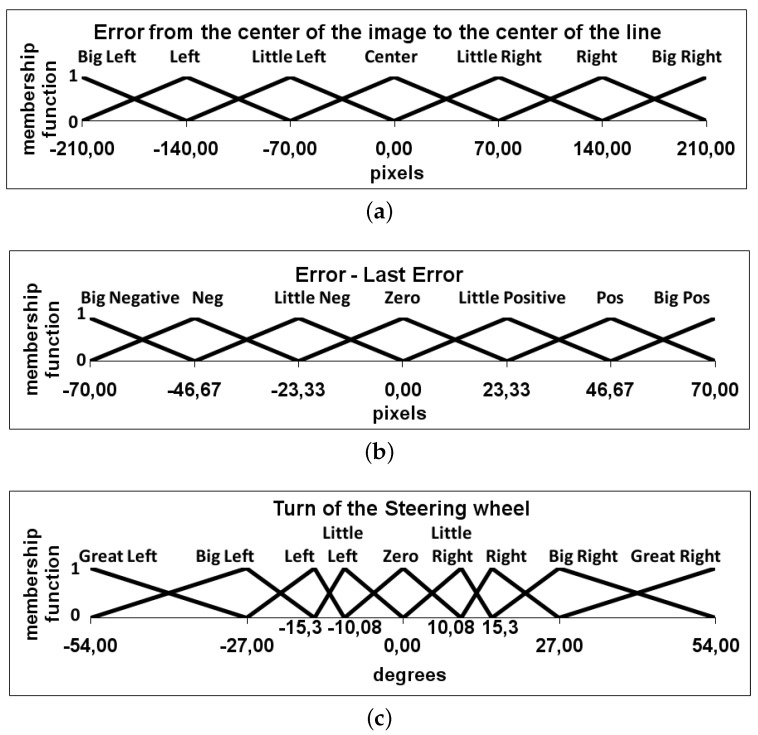
Definition of the two inputs and the output of the fuzzy controller. (**a**) First input variable of the fuzzy controller: Difference between the line reference and the measured line position with respect to the center of the image, in pixels; (**b**) second input variable of the fuzzy controller: The difference between the last error and the actual, in pixels; (**c**) Output variable of the fuzzy controller: The steering wheel angle, in degrees.

The fuzzy-logic controller designed with PD-like behavior has two inputs and one output. Triangular membership functions are used for the inputs and the output. The first input is called the error and is the difference between the line reference and the measured line position in pixels, with respect to the center of the image (Figure 7a). The second input is the derivative of this error (Figure 7b). The output of the controller is the absolute turn of the steering wheel in degrees to correct this error (Figure 7c).

The rule base of the presented fuzzy control component is formed by 49 if-then rules. Heuristic information has been used to define the output of each rule as well as for the definition of the range and set of each variable. The developed fuzzy system is a Mamdani-type controller that uses a height defuzzification model with the product inference model as described in Equation (2).

(2) $$y=\frac{\sum_{l=1}^{M}{\bar{y}}^{l}\;\prod_{i=1}^{N}\left(\mu_{x_{i}^{l}}\left(x_{i}\right)\right)}{\sum_{l=1}^{M}\prod_{i=1}^{N}\left(\mu_{x_{i}^{l}}\left(x_{i}\right)\right)}$$

Herein *N* and *M* represent the number of input variables and the total number of rules, respectively. $\mu_{x_{i}^{l}}$ denotes the membership function of the *l*-th rule for the *i*-th input variable. ${\bar{y}}^{l}$ represent the output of the *l*-th rule.

To tune the fuzzy-logic controller, a driving session performed by a human driver at 10 km/h provided the necessary training data to modify the initial base of rules of the controller and the size of the fuzzy sets of its variables. For the definition of the initial fuzzy sets, a heuristic method was used based on the extraction of statistical measures from the training data. For the initial base of rules, a supervised learning algorithm implemented in MOFS has been used. This algorithm evaluates the situation (value of input variables) and looks for the rules that are involved in it (active rules). Then, according to the steering command given by the human driver, the weights of these rules are changed. Each time the output of an active rule coincides with the human command its weight will be increased. Otherwise, when the output differs from the human command its weight will be decreased by a constant. Anytime the weight of a rule becomes negative the system sets the output of the rule to the one given by the human driver.

Since the velocity of the vehicle is not included in the fuzzy controller, this is taken into account by multiplying the output of the fuzzy-logic controller by $\frac{10}{v}$, where *v* is the current velocity of the vehicle. The definition of the numerator value of this factor is based on the velocity, in km/h, obtained during a driving session with a skilled human driver, in which data was acquired to tune the rule base of the fuzzy controller.

The second component of the presented control system is the weighted integral of the error. The objective of this component is to ensure that the error converges to zero in every kind of track. The output of this component follows Equation (3).

(3) $$I_{t}=I_{t-1}+e\times \frac{1}{t}\times Ki$$

Herein *e* is the current error between the center of the line and the center of the image, *t* is the frame rate, and Ki is a constant that appropriately weights the effect of the integrator. In the presented control approach this constant Ki has a value equal to 0.6.

Finally, the third component of the lateral control system is a steering offset component. It behaves like a feedforward controller that offsets the effect of the change of the curvature of the circuit in every different track, updated each time that a new mark has been detected. It is theoretically calculated using the equations of the Frenet-frame kinematic model of a car-like mobile robot [45]. More detailed information about this control component can be found in [46].

## 6. Experiments

**Figure 8:**
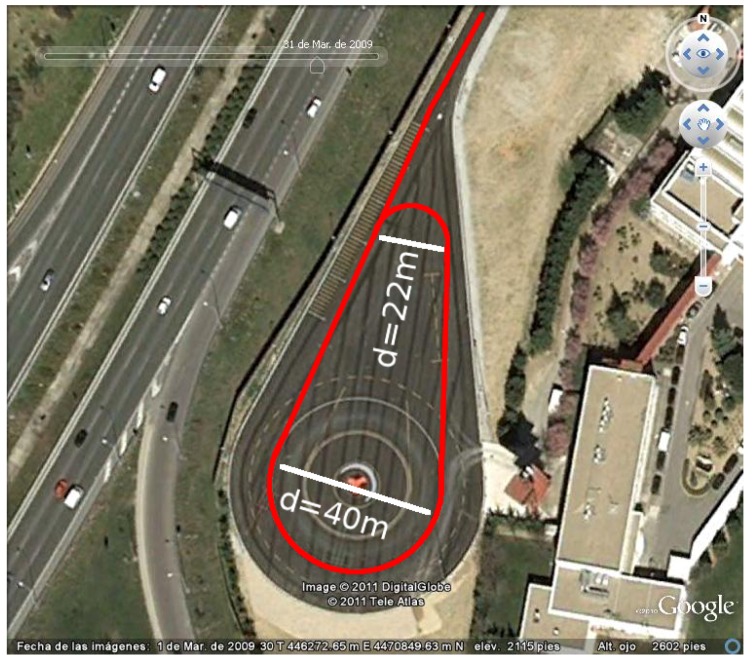
Representation of the circuit at INSIA installations taken with the Google Earth tool.

All the experiments presented in this work were done inside a closed circuit in the infrastructure of INSIA-UPM at the University Institute of Automobile Research, Technical University of Madrid. Figure 8 shows the design of the track. The track has two curves of 11 and 20 m of radius and two straight lines connecting the two curves. A access straight line with a length of 100 m was design to go inside the track and test higher speeds. Each lap of the track has a length of 245 m.

A set of different type of experiments were defined to evaluate the behavior of the full system. Firstly, the experiments related to the computer vision system are presented. Secondly, the different tests to evaluate the autonomous driving and the speed assistance systems are shown.

### 6.1. Computer Vision System

The robustness of the computer vision system was tested in different kind of experiments. Firstly, is presented the robustness of the system against different type of line occlusions. Secondly is presented the behavior of the system against visual marks occlusions and false detections. For each different experiments a set of capture frames are shown, but it must be taken into account that not all the processed images could be saved and are not shown. This is caused by the computational cost of saving images working at the same time as the image processing algorithm. All the experiments presented in this subsection were tested for 13 laps at the speed of 15 km/h and for six laps at different speed from 14 to 42 km/h. During all these experiments the car was drove autonomously. All the occlusions, fork lines, and false detection were overpassed one time for each lap. A slight increase of the RMSE was noticed with respect to similar tests without the occlusions. However, the vehicle complete successfully the 13 at constant speed and the six laps with variable speed without loosing the control in any moment. A comparison table of all the experiments done included the presented on this subsection are shown in the Subsection 6.2.

#### 6.1.1. Line Detection Robustness

The robustness of the vision algorithm to detect the guided line was tested in different situations. Here, it is presented the behavior of the system against a total occlusion of the line, reduction of the width of the line, and presence of other lines.

##### Total Occlusions

A total occlusion is defined as the complete occlusion of the line for a certain distance. The robustness of the system against total occlusion was done to evaluate how much time the system could drive autonomously in blind conditions due to the non-detection of the line. This could be caused by external elements or the complete degradation of the paint of the line itself. Different sizes of occlusions were installed inside the circuit on straight lines and curves.

**Figure 9:**
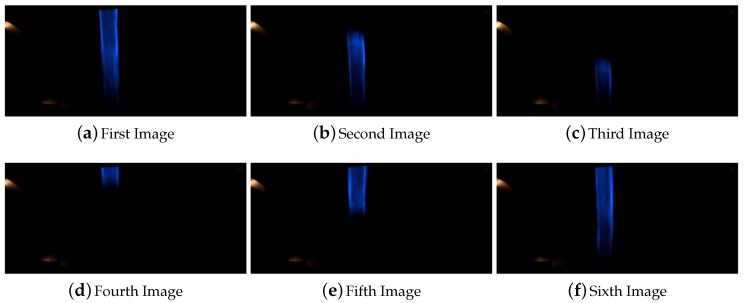
Sequence of images for the line occlusion for 30 cm inside a straight line.

As was explained in Section 4 due to the limitations of the computer vision system the algorithm lost a maximum of 17 cm when the vehicle is driving at the maximum speed of 50 km/h. Here a higher occlusions equal to 30 and 50 cm inside a straight line were tested. Depending on the vehicle speed and when the frames are captured the line can be completely covered and can not be detected for at least one frame in the case of the 30 and 50 cm occlusions. In the presented experiment for a occlusion of 30 cm at 15 km/h a small section of the line was detected in all the frames. In Figure 9 it is shown all the frames captured by the computer vision system during the overpassing of this occlusion.

**Figure 10:**
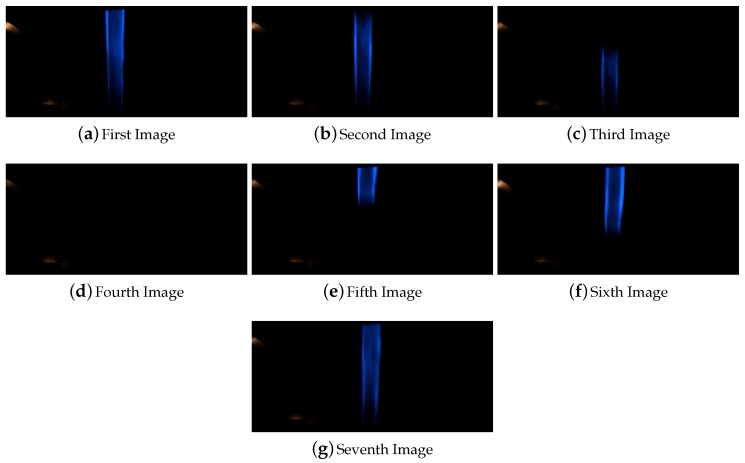
Sequence of images for the line occlusion for 50 cm inside a straight line.

In the case of 50 cm inside straight line the computer vision system lost completely the line for at least one frame, and the occlusion was saw for at least five frames. The Figure 10 shows the images captures during the execution of this test.

**Figure 11:**
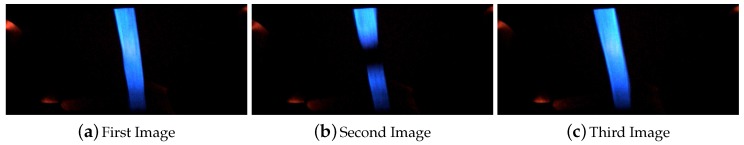
Sequence of images for the line occlusion for 10 cm inside a curve.

**Figure 12:**
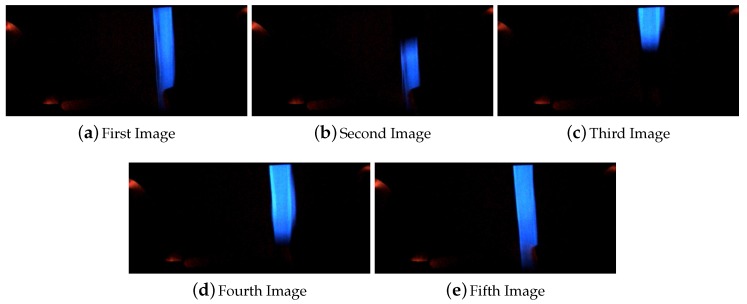
Sequence of images for the line occlusion for 30 cm inside a curve.

The behavior of the system for line occlusions inside curves was also tested. In this case the system was successfully tested for occlusions of 10 and 30 cm. Higher distance occlusions in curve affect the system behavior and could not be 100% certified that the system could operate in satisfactory conditions. Figure 11 shows the images captured for the test of the 10 cm occlusion and Figure 12 shows the images captured during the test of line occlusion for 30 cm.

Both occlusions are located consecutively. The last frame in Figure 12 is the precedent image of the first one in Figure 11.

Higher occlusions than 50 cm inside straight lines and 30 cm inside curves were tested without 100% of success, being them handled by the system only in some specific situations. Based on the presented tests a safety measurement was included to do an emergency stop in case the line was lost for 1 m. This value could be increase or decrease based on the safety requirements of the final system.

**Figure 13:**
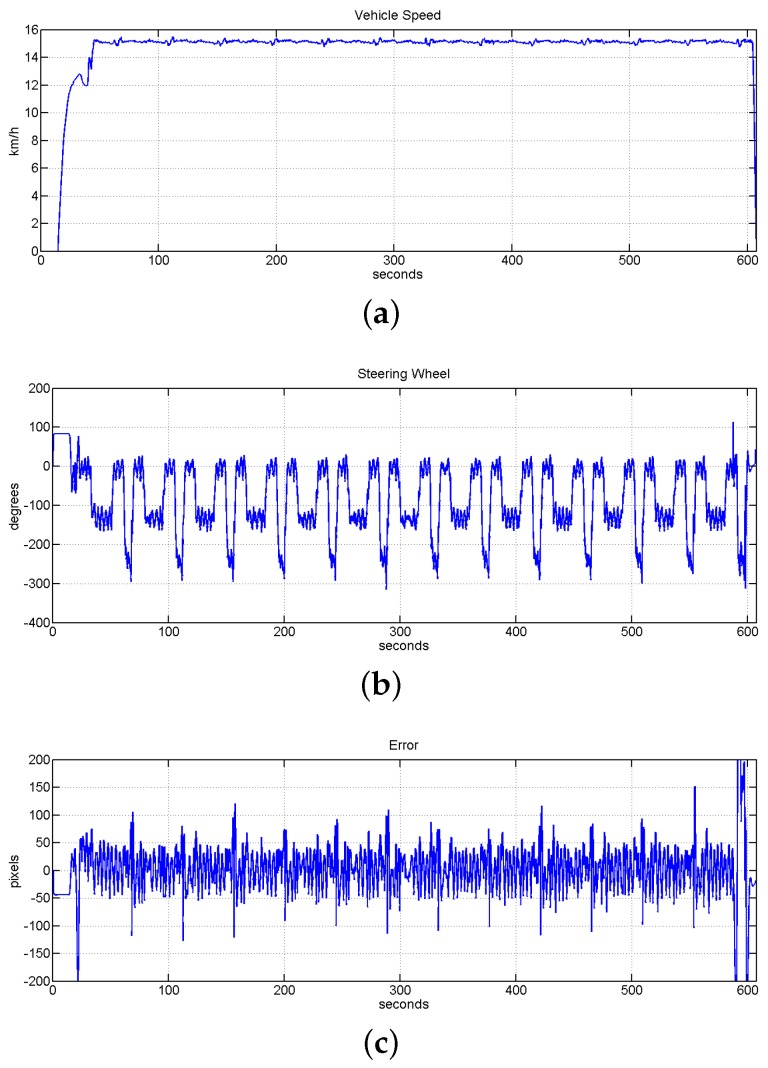
Evolution of the system during 13 laps inside the track with mark detection and different line occlusion onside straight line and curves. Speed was set to 15 km/h. (**a**) Vehicle speed evolution; (**b**) evolution of position of the steering wheel; (**c**) evolution of the error measured in pixels, the value of RMSE for this test is equal to 5.8098 cm.

Figure 13 shows the evolution of the system when all the mentioned occlusions were included in the track for a constant speed equal to 15 km/h. As is shown in this Figure, the autonomous lateral control system managed to command the vehicle without loosing the line and only increasing slightly the total RMSE value to 5.8098 cm in comparison to other similar tests without occlusions, which are presented in Section 6.2.

##### Line Width Reduction

**Figure 14:**
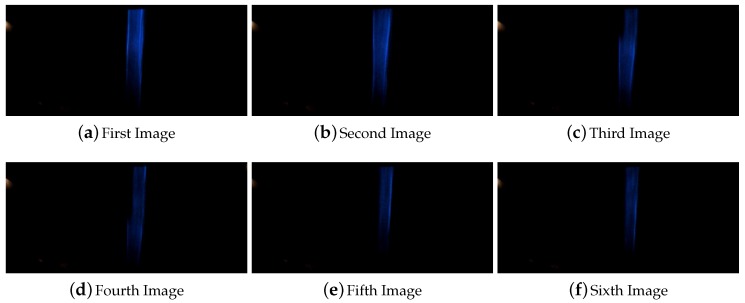
Sequence of images for the reduction of the line width to 3.5 cm.

**Figure 15:**
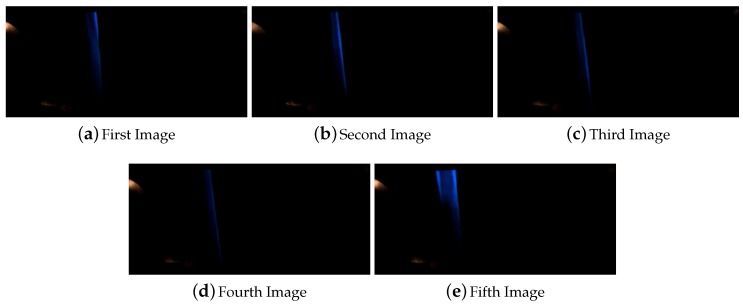
Sequence of images for the reduction of the line width to 2.5 cm.

In this experiment, the line width was set based on the available equipments to paint the line on the road. It was painted manually and the width was in between 5 to 6 cm. Here is presented the experiments done to check the robustness of the system facing thinner lines. The reduction of the width of the line could be caused by a partial occlusion of it, the degradation of the line or the presence of paddles. Furthermore, another objective of these experiment is to evaluate the system behavior with different width of the line for a future real application of the system. A reduction of the 25% and 50% of the line width were applied in a section were the width was equal to 5 cm. Figure 14 shows the images captured for the reduction of the line to 3.5 cm which corresponds to a reduction of the 25% of the width. The captured images of the line shown in Figure 15 corresponds to the overpass of the vehicle over a line with a width reduction equal to the 50% which is a width of 2.5 cm. Furthermore, in both cases it was also possible to see the high degradation of the line that makes this line almost not visible for human eyes but was successfully detected by the system.

The presented line width reduction experiments were included in the circuit for the previously mentioned test of 13 laps shown in Figure 13. For each lap of this test the each line width reduction was overpasses one time.

##### Multiple Lines Simultaneously

In a potential real application of this system it could happen that the computer vision algorithm have to deal with the detection of more than one line simultaneously. That situation could be generated by the presence of previous painted lines, crossroads, or branch roads were two or more lines become one or the main road is divided in two or more lines.

**Figure 16:**
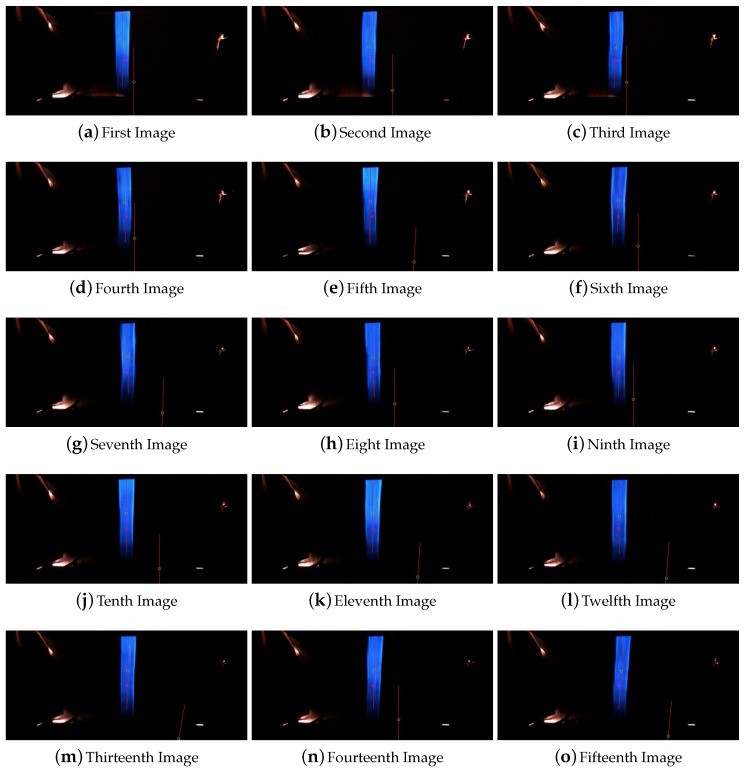
Sequence of images captured with a rejected false positive line.

To avoid to follow other detected lines which are not the current one a voting system between all the detected lines was implemented, as it was previously mentioned in Section 4.1. This system used a temporal information obtained related to the position inside the image of the detected line in the past frames. This system helps to reject false positives and other lines detected. The quality of all the detected lines is also taken into account to identified potential old painted lines. Figure 16 show a sequence of images in where two lines are detected and one of them is rejected as false positive.

Furthermore, the computer vision algorithm rejects all the lines which have an inclination of more than 45° with respect to the vertical of the image frame. This assumption was taken to avoid problems with the presence of other lines in perpendicular direction that could be found in a crossroad situation, when the system overpass other tracks.

**Figure 17:**
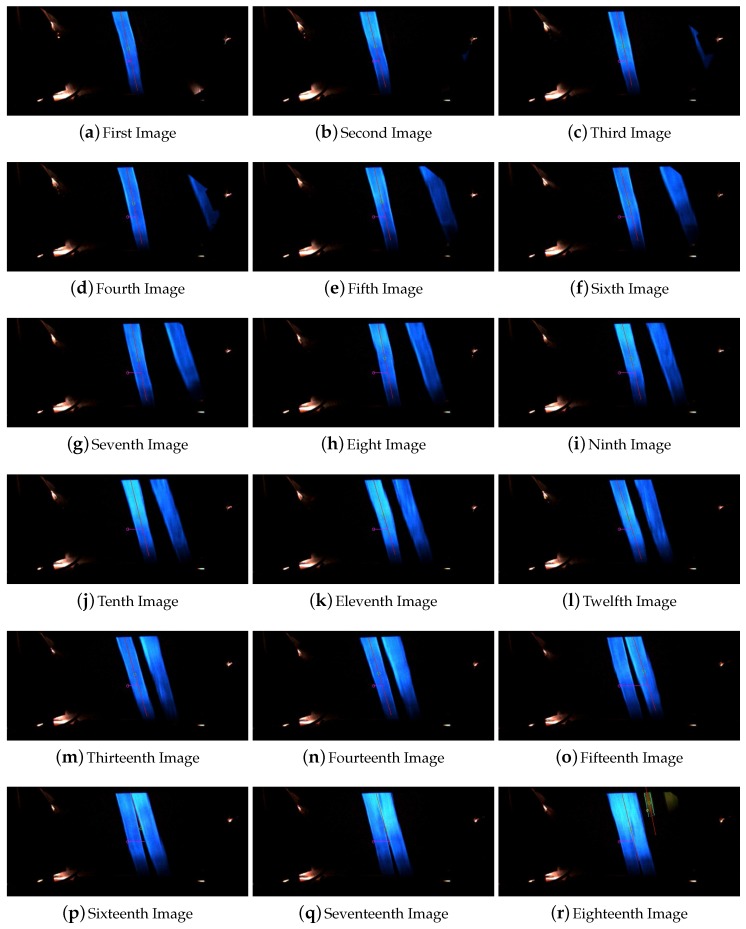
Sequence of images captured overpassing a fork or branch road.

Another situation that was studied in this work is the presence of branch or fork roads. Train systems use this kind of roads to modify the current trajectory of the system to a new track or simply to send it to a garage or specific place for storage or repair the system. Based on this idea we assume that two different situations could happen, one, when the main guided line is divided in two or more, and the other, when the system overpass the junction of two or more lines which become one. Both situations are handle in the same way. An extra information was codified in the visual marks related to it. This information shows if there is or not a fork in this road, and if so where is it, and which of the line has to be track. The present implementation of the system can handle the division of the guided line in three new lines. The value of the variable which manage this situation could be *left*, *center*, *right*, and the system will keep tracking the line on the desired location. This variable could be also modified in real time. Figure 17 shows an example of the second situation in where a fork is overpassed and two lines become one.

#### 6.1.2. Visual Marks Detection Robustness

##### Visual Marks Detection under Partial Occlusion

**Figure 18:**
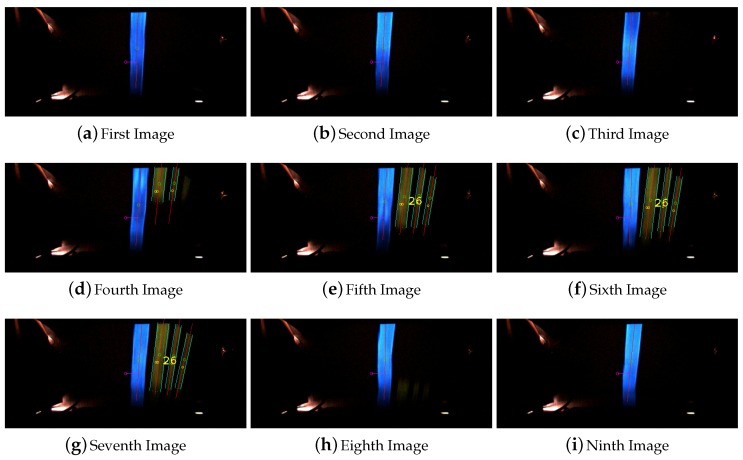
Overpassing and detecting a half-size visual mark at the speed of 15 km/h.

The robustness of the system in relation to the visual marks detection and its potential occlusions was also tested in this work. The defined size of the visual marks is 100 cm. A occlusion of the 50% of the visual marks was tested. The occluded visual mark was integrated in the same experiments as the line occlusions which were presented previously in Figure 13. The occluded visual mark was overpassed at the speed of 15 km/h and it was successfully detected during the 13 laps. One of this detection is shown in Figure 18.

##### False Detection: Wrong ID Identification

**Figure 19:**
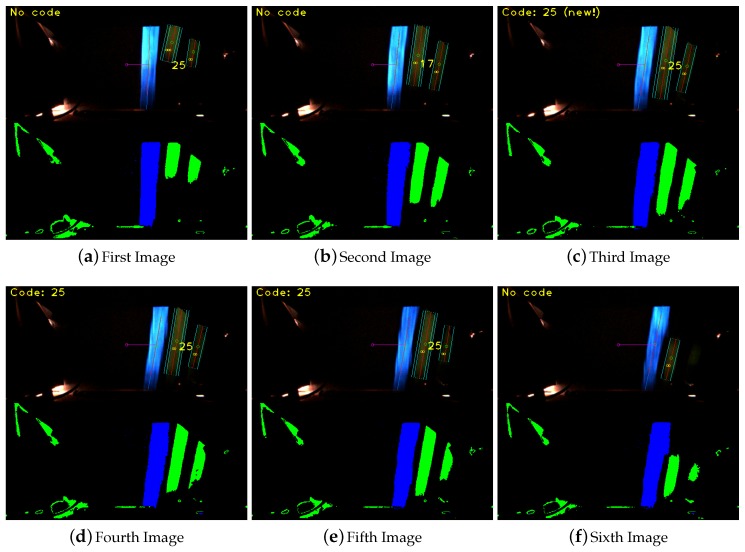
Successful visual mark identification with a false detection of the visual mark 17.

The robustness against false detection of visual marks is done based on two different voting process which was presented in Section 4. These two voting processes check the visual mark in different sections of each frame and based on the winning code detected in the previous frames. Figure 19 shows how the system initially identify the mark 25, then the visual mark 17 and after the mark id 25 again. The final detection and identification is not done until the third frame, when it is mentioned at the top left of the image by the message *Code: 25 (new!)*. The label *(new!)* shows that it is the first frame in which the visual mark was identified after the voting processes. This images sequence also shows the processed image on the bottom of each frame.

##### False Detection: Wrong Detection

**Figure 20:**
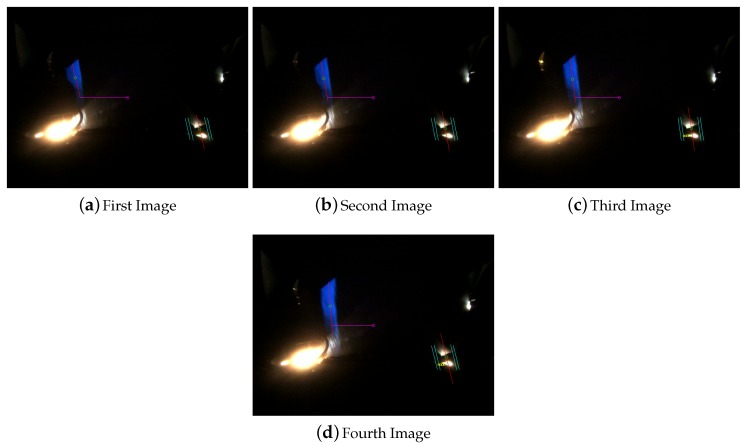
Visual mark wrong detection filtering.

A false detection of a visual mark could be also caused by the detection of a code when there is no mark painted in the road. This could be caused by the presence of light reflections. These reflections are usually happen during the sun dawn and sunset, because the sun rays hit the road more horizontally and could enter into the metal structure where the camera is located. As is shown in Figure 20 the system could detect the sun reflections as visual marks but the codification system and the location definition of the marks, next to the guide line, make the computer vision system robust against this type of false detections too.

#### 6.1.3. Line and Visual Marks Detection at Different Speeds

There were no problems in the detection of the line at different speeds in the interval from 0 to 50 km/h. As set of experiments at different speeds are shown in Section 6.2.

#### 6.1.4. Line and Visual Mark Degradation

The sunlight, rain and other weather factors can deteriorate the paint of the line and the visual marks, decreasing the quality of it and the pixels detected by the system. To detect the degradation process of the line and the marks, the system is evaluating in every captured frame the quality of it based on the number of pixels detected and the color values in predefined threshold intervals. These threshold intervals were set taking into account the acquired values on the experiments done in different weather condition and seasons during the set-up of the system.

### 6.2. Autonomous Driving

In this subsection it is presented the different experiments done to evaluate the behavior of the autonomous driving and speed assistance systems and the robustness of them.

#### 6.2.1. System Stability

Initially, step response tests were done to check the robustness of the control approach against disturbances. These tests were defined changing the desired position of the line by 50 pixels. These step disturbance’s tests were done in straights and curves at the different speeds of 10, 15, and 20 km/h. Table 1 shows the results of all the tests performed and the value of the Root Mean Squared Error (RMSE) for each test, which never were bigger that 8 cm.

#### 6.2.2. System Robustness

Once the system stability was tested, we checked the robustness of the system by different type of experiments. The results of the most relevant tests are shown in Table 2. This table is divided by the type of experiments into six different blocks. Following, it is mentioned separately the details of each type of experiments including the graphs of some of them. It has to be taken into account that the RMSE value is calculated from the beginning of the test until the end of it. Slightly different values of the RMSE for similar tests could be calculated based on the different acceleration/brake actions.

##### Final Control Adjustments

**Figure 21:**
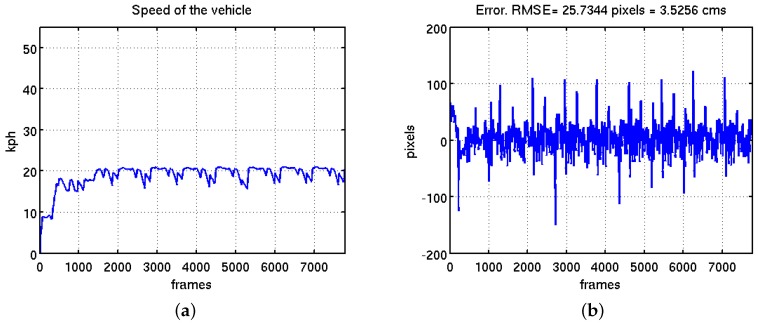
Finest adjustment of the steering wheel control system. (**a**) Vehicle speed evolution; (**b**) evolution of the error in pixels with a RMSE equal to 3.52 cm.

The first block shows the experiments done for the finest adjustment of the control system at different speeds between 10 and 20 km/h. Basically the part of the controller that was adjusted in these tests was the gain of the integral part. The gain adjustment was done in real time, testing one different value per each lap. A post-evaluation of the results and the error graphs were done to set-up the final value of the gain. The Figure 21 shows Experiment 2 mentioned in Table 2. In Figure 21b is shown the evolution of the error and slight differences for each lap.

##### Long Distance Robustness

**Figure 22:**
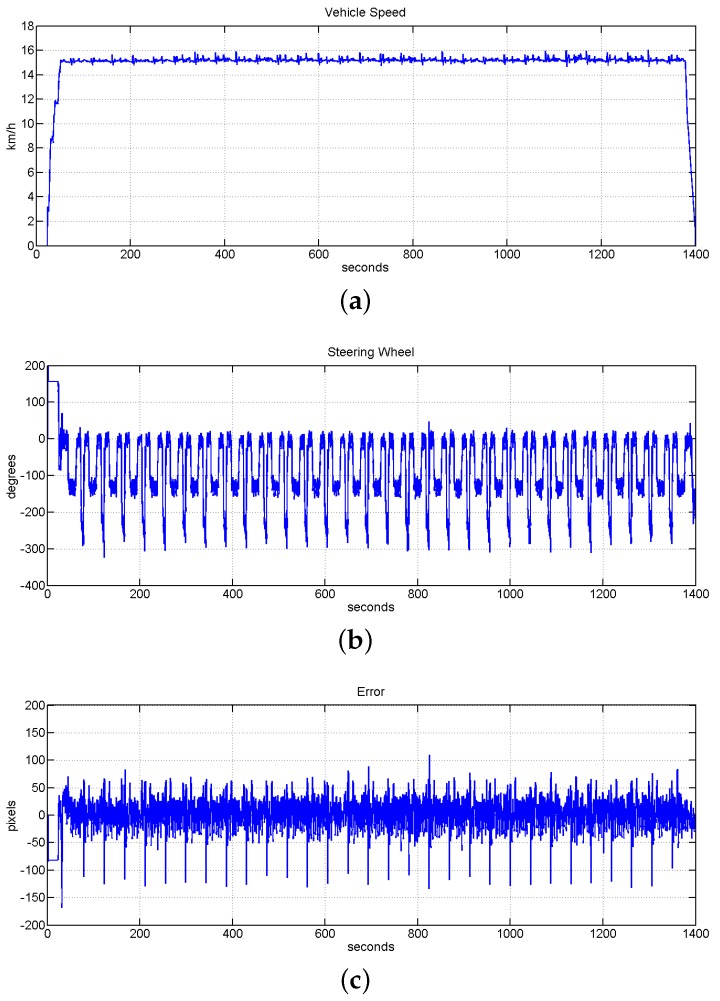
Evolution of the system to check the robustness of the system for a long distance with a reduced speed value. (**a**) Vehicle speed evolution; (**b**) evolution of the steering wheel; (**c**) error measured (in pixels), the value of RMSE for this test is 3.6874 cm.

Once the control system was completely set-up, the robustness of the system for long distances at low speed was tested. In this case the speed of the system was set from 10 to 16 km/h. The system was successfully tested in covering a distance of 7.3 km, which is equivalent to 30 laps of the circuit at the speed of 15 km/h and 21 laps at different speed between 10 and 16 km/h. The system accomplished successfully both experiments with a RMSE error smaller than 4 cm. The experiment in which the vehicle was covering the distance of 7.6 km with the autonomous driving and assistance speed system working together is shown in Figure 22. In this Figure it is shown the evolution of the speed value, the evolution of the position of the steering wheel and the error for the experiment of 30 laps. In this case the RMSE of the full tests was reduced to 3.6874 cm. Analyzing the plot shown in Figure 22b the localization of the vehicle inside the track can be intuited, being the vehicle in a straight line when the steering wheel is around 0 degrees. The curve of 11 m of radius is represented by the maximum turn of the steering wheel around 250 degrees, and the bigger curve with 20 m of radius is represented by the small turn around 150 degrees.

##### Line/visual Marks Occlusions

The third block of experiments are related to the evaluation of the system when there are line and visual marks occlusions. All these experiments were presented in the previous Section 6.1.

##### Speed Limits

**Figure 23:**
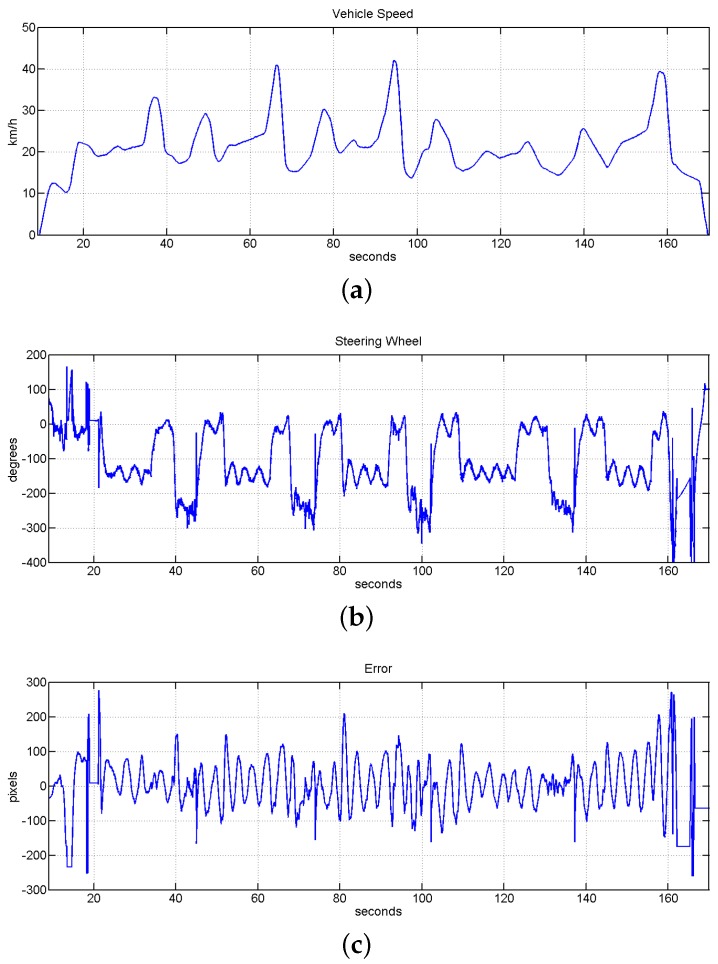
Evolution of the system under speed limits with human speed control experiment. (**a**) Vehicle speed evolution with a top speed of 42 km/h; (**b**) evolution of the steering wheel position; (**c**) evolution of the error measured in pixels, the value of RMSE for this test is equal to 9.1397 cm.

The fourth block of experiments was designed to test the speed limits of the system under human speed control. In this case the speed assistance system was not active and a human was controlling the gas and brake pedals. Here is also checked the behavior of the system against faster accelerations and strong breaks. Table 2 shows two different experiments inside this block in which the speed was modified from 6 to 38 km/h and from 14 to 42 km/h. Figure 23 shows the evolution of the system in one of these tests. It is also checked in this test the behavior of the system when the vehicle starts non correctly aligned with the guided line. This could be checked in the first 20 s in Figure 23b,c by initial strong movements of the steering wheel and high error values.

##### Speed Assistance

In this block of experiments it was tested the performance of the speed assistance system. As is shown in the previous block of experiments, here it is also tested the speed limits of the system, but in this case we focus this action on the curves. The stop and restart of the vehicle based on visual mark detections was also tested here. These stops/restart actions try to emulate the stop of a public transport and were checked in curves and straights.

**Figure 24:**
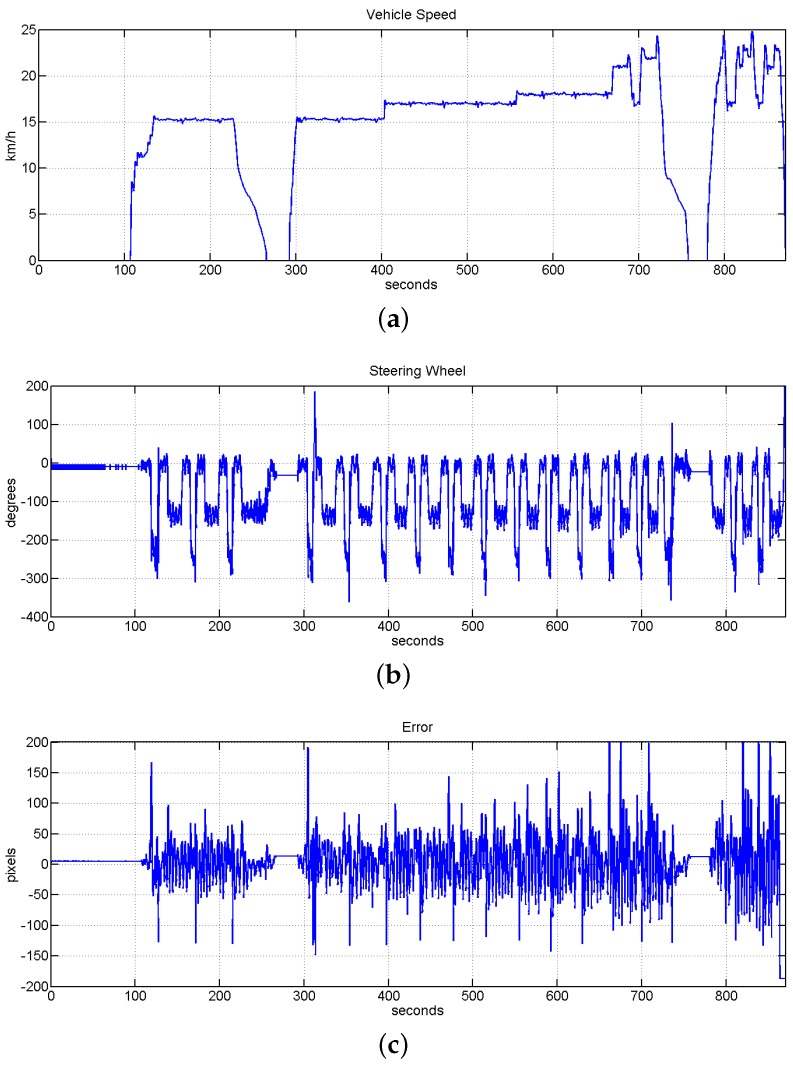
Evolution of the system with the speed assistance and the autonomous driving system working together and checking speed limits. It is also shown the response of the system to accomplish visual marks based stops. (**a**) Vehicle speed evolution; (**b**) evolution of the steering wheel; (**c**) evolution of the error measured in pixels, the value of RMSE for this test is equal to 5.5535 cm.

**Figure 25:**
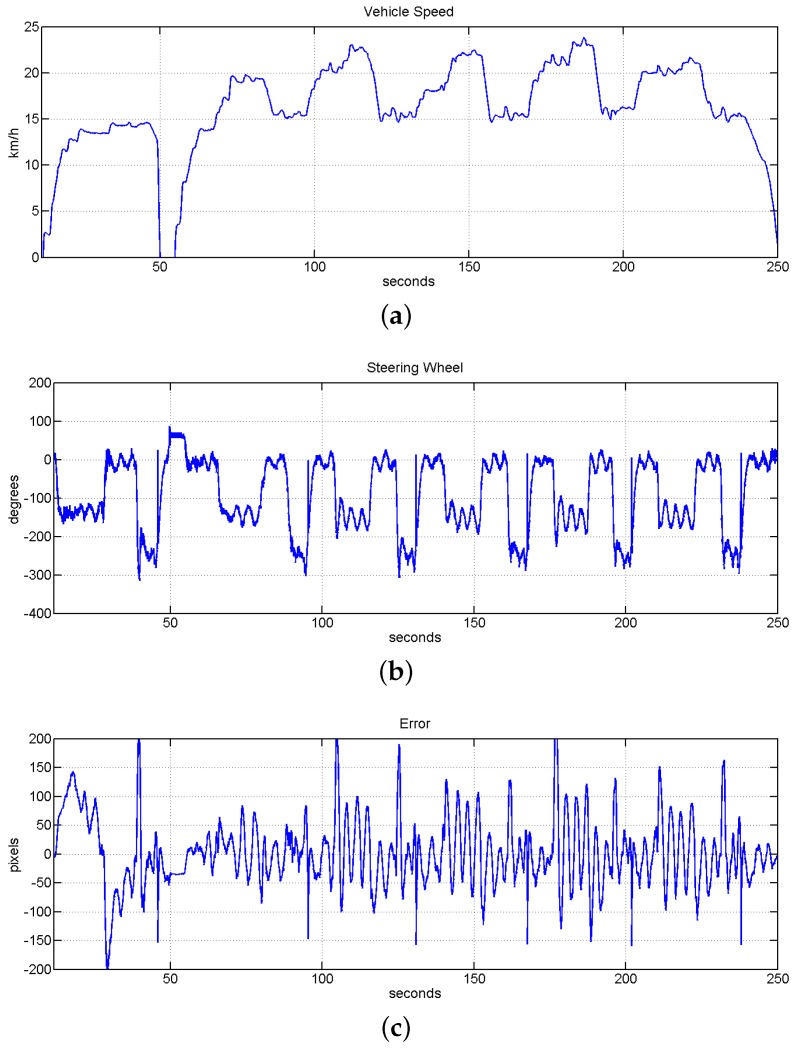
Evolution of the system with the speed assistance and the autonomous driving system working together. It is also shown the response of the system when a emergency stop is detected and done. (**a**) Vehicle speed evolution; (**b**) evolution of the steering wheel; (**c**) evolution of the error measured in pixels. The value of RMSE for this test is equal to 8.4839 cm.

The behavior of the speed assistance system was also tested to response with an emergency stop of the vehicle. Here it is emulated the emergency situation by the detection of one specific visual mark. The system was configured to push the brake pedal until the end with the consequent strong deceleration. Figure 24 shows the behavior of the system on the emergency stop and also the evolution of the system when the speed is controlled by the speed assistance. In this test is also possible to see how the different sections are set with different speeds and how the speed assistance control it.Figure 25 shows another test of the emergency stop with the speed controller manually by the driver.

During the experiments presented on this and the previous blocks the comfort of the vehicle respect to the speed and the behavior of the lateral control system were tested at different speeds. The evaluation of these tests was done based in the feedback of some participants and the examination of the data obtained. Finally the speed limits of each section were set based on the extracted information.

##### Top Speed

**Figure 26:**
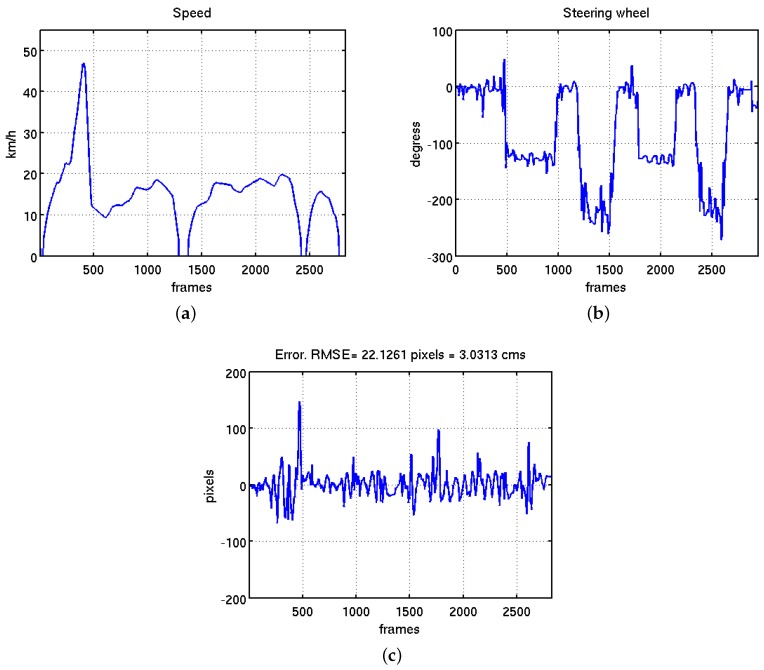
Evolution of the system under top city speed of 50 km/h and big speed changes. (**a**) Vehicle speed evolution with a top speed of 48 km/h; (**b**) evolution of the steering wheel; (**c**) evolution of the error measured in pixels, the value of RMSE for this test is equal to 3.0313 cm.

The last block of tests focus on the robustness of the controller against big speed changes. In this test it is also checked the response of the system when the vehicle is traveling close to the maximum speed allowed in inner-city environment (50 km/h). In this case the speed was controlled by a human to applied a strong acceleration that permits to reach the maximum speed in the available straight access of the circuit. As is shown in Figure 26a, the autonomous vehicle reaches a top speed of 48 km/h. After this, a strong reduction of the speed was applied to enter in the biggest curve. Two stop/restart actions inside the smallest curve were also tested in this experiment. Figure 26c shows the error during these tests. The line was not lost in any of this strong stops and accelerations and the RMSE value along this experiment was equal to 3.0313 cm. This value obtained represents just the 6% of the field of view of the perception system. The movements of the steering wheel commanded by the fuzzy controller is shown in Figure 26b.

The robustness of the system against line loss had been also tested and it is presented in details in [47]. In this previous work, no mark information was given to the control system with a maximum speed reduced. Successful results had been obtained following the same track without any information with a average speed of 15 km/h during more than 15 laps covering more than 4 km. Some tests of step commands in straight and curves at 10 and 15 km/h are shown too.

A video of the system working is available online [48].

## 7. Conclusions and Future Work

The presented work shows a vision-based control system approach to command the steering of a passenger vehicle to follow a route. Furthermore, an assistance speed system and an approach to localize the vehicle inside the route based on a single vision sensor is also presented. The vision sensor was located inside a metal structure with a limited size of 30×50 cm. The definition of the path to follow was done by a line painted on the floor using ultraviolet sensible paint. The information measured by the computer vision system using the painted line was used as an input by a mixed control system that commands the steering of the vehicle. This control system is formed mainly by a PD-like fuzzy-logic controller, as well as a feedforward controller whose inputs are the position of the vehicle in the circuit and the curvature of the current section of the circuit. This extra information is also provided by the computer vision system by means of visual marks also painted on the road with a special UV sensible paint. Furthermore, these visual marks provide information related to the speed limit of the vehicle in each section of the route. The full system was successfully tested in a real passenger car inside a closed circuit traveling for more than 7 km without a driver and any interruption. Furthermore, different experiments were presented to test a maximum speed of 48 km/h, emergency stops and strong speed changes. The robustness of the computer vision algorithms against line and visual mark occlusions, false detection and the presence of more lines was also presented in a set of experiments. Based on the results of all of the experiments presented in this work, the presented approach is able to command a vehicle in inner-city environments as public transport without taking into account the obstacle avoidance. This is in fact a very important point in which the authors are focusing their effort. Another important part that could be improved is the dependence of the vision structure to avoid the illumination changes. The authors are also working in parallel in developing vision approaches that could be robust against illumination changes.

## Figures and Tables

**Table 1:** Evaluation of the system behavior against step disturbances of 50 pixels at different speeds and location inside the track.

Step Size	Circuit	Speed	RMSE
(pixels)	Section	(km/h)	(cm)
50	straight	10	7.5051
50	straight	10	6.8402
50	straight	15	7.8274
50	straight	15	6.7190
50	curve	10	6.9561
50	curve	10	5.1034
50	curve	15	5.5429
50	curve	15	6.5174
50	curve	15	6.2648
50	curve	20	6.9676

**Table 2:** RMSE evaluation of the different experiments done.

Test	Number	Min Speed	Top Speed	RMSE	Detailed Information
id	of Laps	(km/h)	(km/h)	(cm)	
1	9	10	15	4.8270	Small control adjustments
2	13	15	20	3.5256	Small control adjustments
3	21	10	16	3.4100	Long distance robustness with small speed variations
4	30	15	15	3.6874	Long distance robustness at constant speed
5	13	15	15	5.8098	Line/visual-marks occlusions at constant speed
6	6	14	42	10.7916	Line/visual-marks occlusions at different speeds
7	4	6	38	8.6494	Human speed control + testing speed limits of the circuit
8	4	14	42	9.1397	Human speed control + testing speed limits of the circuit
9	6	14	24	8.4839	Speed assistance + testing speed limits for the big curve
10	14	10	23	7.5486	Speed assistance + testing speed limits for the small curve
11	17	15	25	5.5535	Speed assistance + stop/start and diff. speeds
12	2	10	48	3.0313	Top speed + two stop/start in curves
